# The impact of ankle–foot orthoses on toe clearance strategy in hemiparetic gait: a cross-sectional study

**DOI:** 10.1186/s12984-018-0382-y

**Published:** 2018-05-23

**Authors:** Kannit Pongpipatpaiboon, Masahiko Mukaino, Fumihiro Matsuda, Kei Ohtsuka, Hiroki Tanikawa, Junya Yamada, Kazuhiro Tsuchiyama, Eiichi Saitoh

**Affiliations:** 1 Department of Rehabilitation Medicine I, School of Medicine, Fujita Health University, 1-98 Dengakugakubo, Kutsukake, Toyoake, Aichi 470-1192 Japan; 2 Faculty of Rehabilitation, School of Health Sciences, Fujita Health University, Toyoake, Aichi Japan; 3 Department of Rehabilitation, Fujita Health University Hospital, Toyoake, Aichi Japan

**Keywords:** Orthosis, Hemiplegia, Gait, Rehabilitation, Compensation, Swing phase

## Abstract

**Background:**

Ankle–foot orthoses (AFOs) are frequently used to improve gait stability, toe clearance, and gait efficiency in individuals with hemiparesis. During the swing phase, AFOs enhance lower limb advancement by facilitating the improvement of toe clearance and the reduction of compensatory movements. Clinical monitoring via kinematic analysis would further clarify the changes in biomechanical factors that lead to the beneficial effects of AFOs. The purpose of this study was to investigate the actual impact of AFOs on toe clearance, and determine the best strategy to achieve toe clearance (including compensatory movements) during the swing phase.

**Methods:**

This study included 24 patients with hemiparesis due to stroke. The gait performance of these patients with and without AFOs was compared using three-dimensional treadmill gait analysis. A kinematic analysis of the paretic limb was performed to quantify the contribution of the extent of lower limb shortening and compensatory movements (such as hip elevation and circumduction) to toe clearance. The impact of each movement related to toe clearance was assessed by analyzing the change in the vertical direction.

**Results:**

Using AFOs significantly increased toe clearance (*p* = 0.038). The quantified limb shortening and pelvic obliquity significantly differed between gaits performed with versus without AFOs. Among the movement indices related to toe clearance, limb shortening was increased by the use of AFOs (*p* < 0.0001), while hip elevation due to pelvic obliquity (representing compensatory strategies) was diminished by the use of AFOs (*p* = 0.003). The toe clearance strategy was not significantly affected by the stage of the hemiparetic condition (acute versus chronic) or the type of AFO (thermoplastic AFOs versus adjustable posterior strut AFOs).

**Conclusions:**

Simplified three-dimensional gait analysis was successfully used to quantify and visualize the impact of AFOs on the toe clearance strategy of hemiparetic patients. AFO use increased the extent of toe clearance and limb shortening during the swing phase, while reducing compensatory movements. This approach to visualization of the gait strategy possibly contributes to clinical decision-making in the real clinical settings.

**Trial registration:**

UMIN000028946. Registered 31 August 2017 (retrospectively registered).

**Electronic supplementary material:**

The online version of this article (10.1186/s12984-018-0382-y) contains supplementary material, which is available to authorized users.

## Background

Impaired paretic limb advancement is a clearly observable manifestation of gait pathology in individuals with hemiparesis due to stroke [1–3]. Previous studies have reported specific gait changes following hemiparesis, such as decreased knee flexion, hip flexion, and ankle dorsiflexion during the swing phase, which can negatively influence the achievement of toe clearance [1–6]. Reduction in toe clearance of the affected limb leads to tripping while walking, which is a major cause of falls [7, 8]. In healthy individuals, toe clearance is mainly achieved by limb shortening, which is affected by hip flexion, knee flexion, and ankle dorsiflexion. On the other hand, to obtain sufficient toe clearance during the swing phase, individuals with hemiparesis often require compensatory strategies that modify the kinematic pattern, including hip hiking and circumduction, which are common gait deviations [3, 9]. These changes during the swing phase have a reciprocal relationship. When the limb shortening is reduced due to paresis, the compensatory movements will be increased to contribute to toe clearance; hence, they are in a trade-off relationship [10].

Ankle–foot orthoses (AFOs) are frequently prescribed to improve walking ability in hemiparetic patients, as they provide passive or dynamic support of ankle movement. AFOs provide support not only during the stance phase of gait by encouraging lateral stability or improving early stance knee moments, but also in the swing phase to maintain ankle dorsiflexion and facilitate toe clearance [11–17]. The effect of AFOs on the swing phase is additionally reflected in the compensatory movements. Cruz et al. [18] demonstrated that the compensatory pelvic obliquity observed in response to impaired ankle dorsiflexion in hemiplegic patients was minimized when the patients wore an AFO. Improved joint motions and decreased compensatory movement when using AFOs could potentially contribute to an efficient gait and promote walking activity in hemiparetic patients.

Clarification of the mechanical effect of AFOs on these gait parameters, and quantifications of compensatory movements would be helpful for clinical decision-making in rehabilitation clinics. For example, understanding the influence of rehabilitative training and the use of AFOs on gait indices (i.e., ankle angle, knee angle, hip elevation, or toe clearance) would help to determine the best rehabilitative strategy and to identify the need for AFO use in individual patients.

The aim of this study was to clarify the mechanical effect of AFOs and to quantify the impact of AFO use on hemiparetic gait pattern during the swing phase, as this information would be helpful for clinical decision-making in rehabilitation clinics. For example, understanding the influence of rehabilitative training and the AFO and its types on gait indices (i.e., ankle angle, knee angle, hip elevation, or toe clearance) would help to determine the best rehabilitative strategy and to investigate the need for AFO use in individual patients. Based on a prior study showing the relationship between limb shortening and compensatory movements [10], we hypothesized that the AFOs would positively affect functional limb shortening in a way that would consequently impact on toe clearance and compensatory maneuvers, particularly represented by hip elevation. Previous studies have shown the effects of AFOs and a relationship between limb shortening and compensatory movements. In the normal gait pattern, functional limb shortening (representing lower limb joint movement) is a main strategy for toe clearance. However, patients with hemiparesis have impaired lower limb function, and thus require compensatory strategies (e.g., hip hiking, circumduction of the paretic limb) to promote swing phase propulsion [19, 20]. Additionally, the extent of toe clearance is mainly determined by the extent of functional limb shortening and hip elevation as compensatory movements, which are in a trade-off relationship [10]. AFO usage reduces the gait pattern deviation and increases the walking ability, thereby reducing energy costs [21, 22]. In this study, we hypothesized that the AFOs would positively affect functional limb shortening in a way that would consequently impact on toe clearance and compensatory maneuvers, particularly represented by hip elevation. To determine the actual impact of limb shortening and compensatory movements on toe clearance, the vertical component of the movements that comprised toe clearance was calculated using three-dimensional kinematic motion analysis. The changes in joint angles were also investigated.

## Methods

### Participants

Twenty-four patients with post-stroke hemiparesis in either the subacute (time after onset; TAO ≤ 90 days) or chronic (TAO > 90 days) stages who received rehabilitation training at the Fujita Health University Rehabilitation Complex Center were recruited for this study. The study participants were 18 males and 6 females aged 47 ± 19 years (mean ± SD). Eleven patients had right hemiplegia, and 13 had left hemiplegia. The duration of hemiparesis ranged from 1 to 81 months. Nine participants were in the subacute stage, and the remaining 15 participants were in the chronic stage. The participants were evaluated on a range of neurological motor impairments with the stroke impairment assessment set (SIAS). In evaluating the lower extremity, three items including hip flexion, knee extension, and foot tap were tested, and each item was rated from 0 (severely impaired) to 5 (normal) for expressing motor function of lower extremities (maximum score 15) [23]. All participants used their personal AFOs in daily life and had the ability to walk independently on a treadmill without orthoses, handrails, or any assistive devices. The types of AFOs were classified into two groups: thermoplastic AFOs (tAFOs) or adjustable posterior strut AFOs (APS-AFOs). The APS-AFO is an articulated AFO that is used for gait rehabilitation in hemiparetic patients [24–26]. The APS-AFO allows easy adjustment of the ankle-hinge joint, as the length and thickness of the strut can be changed to suit the patient (Additional file 1: Figure S1). Half of the participants (12 patients) used tAFOs during the walking trial, and the other half used APS-AFOs. Patients were excluded if they marked cardiorespiratory or metabolic disease, history of previous neuromuscular diseases or orthopedic conditions that may limit walking ability, or impaired cognitive or communicative ability to follow instructions. This study was approved by the Medical Ethics Committee board of Fujita Health University. All patients provided written informed consent prior to participation.

### Procedure

Kinematic data was acquired via three-dimensional treadmill gait analysis performed using a simplified gait analysis system (KinemaTracer^®^; Kissei Comtec Co., Ltd., Matsumoto, Japan). The KinemaTracer® system is composed of a computer for recording and data analysis, and four charge-coupled device cameras with 60 Hz frame rates installed around both sides of the treadmill. The measurement error for this system was determined using a modified protocol based on the evaluation protocol of measurement error developed by The Clinical Gait Analysis Forum of Japan [27]. The averaged absolute error for each axis ranged from 0.5 to 2.4 mm, which is comparable to existing systems [27, 28] (Additional file 2: Supplemental methods and Additional file 3: Figure S2).

**Fig. 1:**
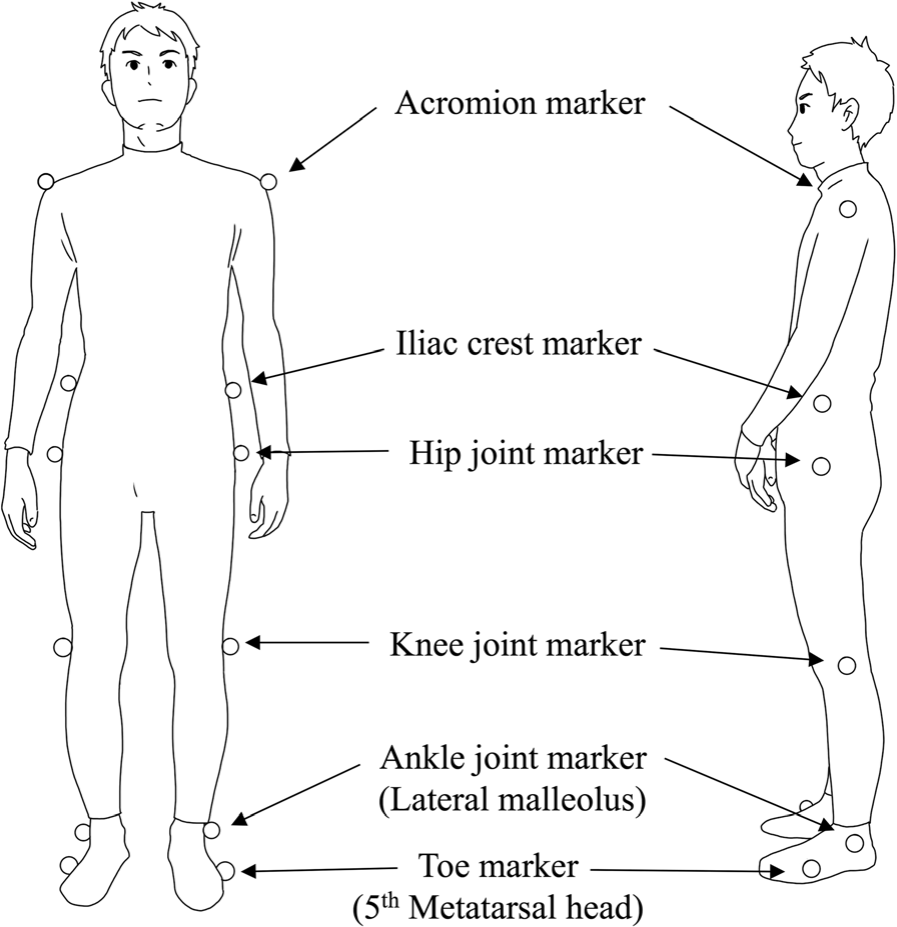
Marker placement. The positions of 12 measurement markers (bilateral acromion, iliac crest, hip, knee, ankle and toe)

A total of 12 markers (30 mm in diameter) were placed bilaterally on the acromion processes, iliac crests (on a vertical line passing through the hips), hip joints (at points one-third from the greater trochanter on the line between the greater trochanter and the anterior superior iliac spine), knee joints (on the midline of the anteroposterior diameter of the lateral epicondyle of the femur), lateral malleoli, and the fifth metatarsal heads (Fig. 1). Although the first toe is more commonly used to put the toe marker, the fifth metatarsal heads are selected in this study, for the following reasons: the marker tracking with this system would be more stable with the markers placed on the 5th metatarsal head than on the 1st metatarsal head. The foot marker at the 5th metatarsal head also will better reflect the real floor-to-floor clearance (toe clearance) in patients with equinovarus, which is frequently seen in hemiparetic patients. The feasibility of this method in real clinical settings has been verified in previous studies [29, 30]. All participants practiced walking on the treadmill until they became accustomed to it. A rest interval of 5 to 10 min was provided prior to test initiation. Each patient was then asked to walk at a comfortable self-selected speed with and without their AFO. To reduce the variability in kinematic adaptations, we applied the same process for all the patients to select the speed: 1) The ground gait speed without AFO was measured during a 10 m walk. 2) The treadmill speed was set at 70% of that ground gait speed. 3) If the patients did not feel comfortable, the speed was gradually increased until the patients felt comfortable at the maximum of the ground gait speed.

**Fig. 2:**
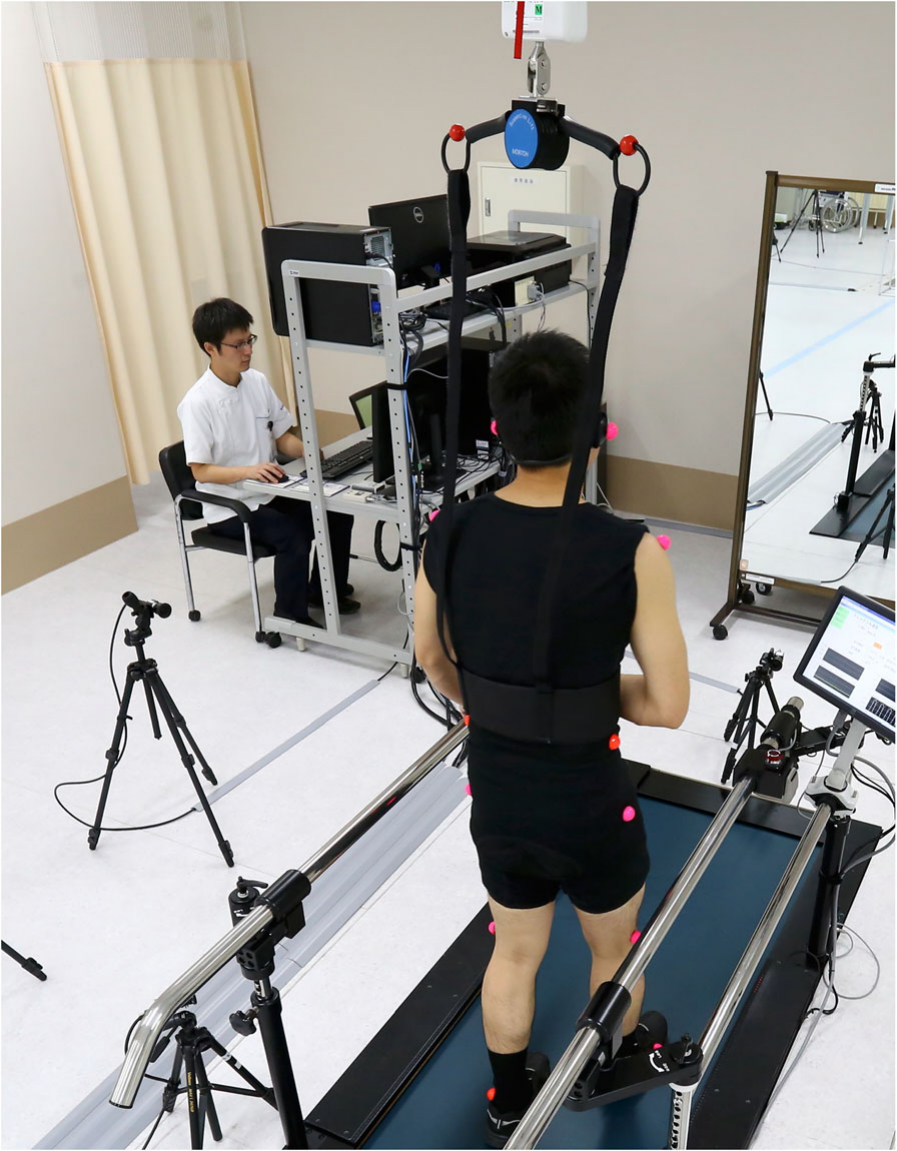
Measurement using the simplified gait analysis system. The patients walk on the treadmill for the measurement. Safety suspension and a handrail are provided to prevent falls during the measurement

The order of these two conditions (with or without an AFO) was selected randomly. The appropriate footwear was prescribed to match the AFO for each patient during trials performed with and without AFOs. There was a 5-min rest period between trials. Additional assistive devices or handrails were not allowed during the walking trial. The duration of the gait measuring time was 20 s, and data capture was started when patients achieved a steady state walking speed in each trial. The average number of gait cycles was 16.7 ± 1.8 without the AFO and 16.3 ± 1.9 with the AFO. A single measurement session took about 20 min to complete, making this a feasible procedure for use in a real clinic. During each measurement, a technician managed the measurements and treadmill controls and a physical therapist stood by to supervise the patient and to prevent falls. Safety suspension and handrail was also prepared (Fig. 2). Additional procedures for checking marker tracking errors, when necessary, required 10 to 20 min for each patient.

### Outcome measures and statistics

**Fig. 3:**
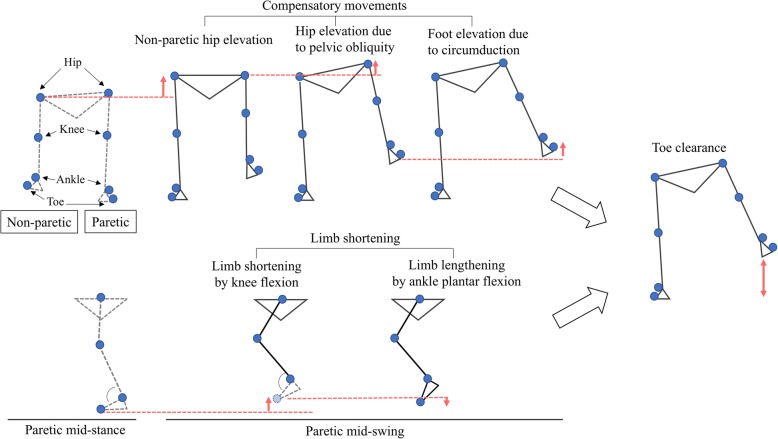
Schematic diagram of analysis of the components of toe clearance. The two components of limb shortening (limb shortening by knee flexion and limb lengthening by ankle plantar flexion) and the three components of compensatory movements (non-paretic hip elevation, hip elevation due to pelvic obliquity, and foot elevation due to circumduction) are visualized. All indices were calculated from the difference in the vertical position of the markers between the paretic mid-stance and the paretic mid-swing

The present study calculated the following as indices of lower-limb function and compensatory movements of the paretic limb during the swing phase: toe elevation from the walking surface, the vertical component of functional limb shortening in terms of hip-toe distance, hip elevation due to pelvic obliquity, non-paretic hip elevation, and foot elevation due to foot lateral shift (circumduction). The mid-swing values were calculated at the time point at which the swing toe of the paretic side crossed beneath the hip marker on the sagittal plane. At this time, the vertical component of the lateral foot shift by circumduction could be calculated independently of the limb movement on the sagittal axis, without considering the effect of the inclination of the hip-toe line on the sagittal plane. The mid-stance values were calculated at the time point at which the stance toe of the paretic side crossed the vertical line drawn from the hip marker on the sagittal plane. All the indicators of dynamic movement were calculated with respect to the vertical direction (Fig. 3). All values were calculated using an automated process.

### Indices for limb shortening

Limb shortening was divided into two components: limb shortening due to knee/hip joint movement and limb shortening or lengthening due to ankle joint movement. The limb shortening due to knee joint movement was derived by calculating virtual limb shortening assuming the ankle joint angles to be fixed. The limb shortening or lengthening was derived by calculating the difference between the actual total limb shortening and the limb shortening due to knee joint movements.

### Indices for compensatory movements

Hip elevation due to pelvic obliquity, non-paretic hip elevation, and foot elevation due to foot lateral shift (circumduction) can be understood as movements compensating for toe clearance, because these movements are not observed in normal gait patterns [10].

Hip elevation due to obliquity on the paretic side was calculated from the vertical movement of the hip marker on the paretic side, which was taken to represent hip hiking. Non-paretic hip elevation, which is seen in hemiplegic patients with severe lower-limb dysfunction [31], was calculated as the difference in vertical position of the non-paretic hip marker between the mid-stance and the mid-swing of the paretic limb. The foot elevation due to foot lateral shift was calculated from the vertical value due to the displacement of the lateral malleolus.

### Index of toe clearance

The elevation in the vertical axis of the fifth metatarsal heads from the walking surface was measured as an indicator of toe clearance.

### Calculation of joint angles

**Fig. 4:**
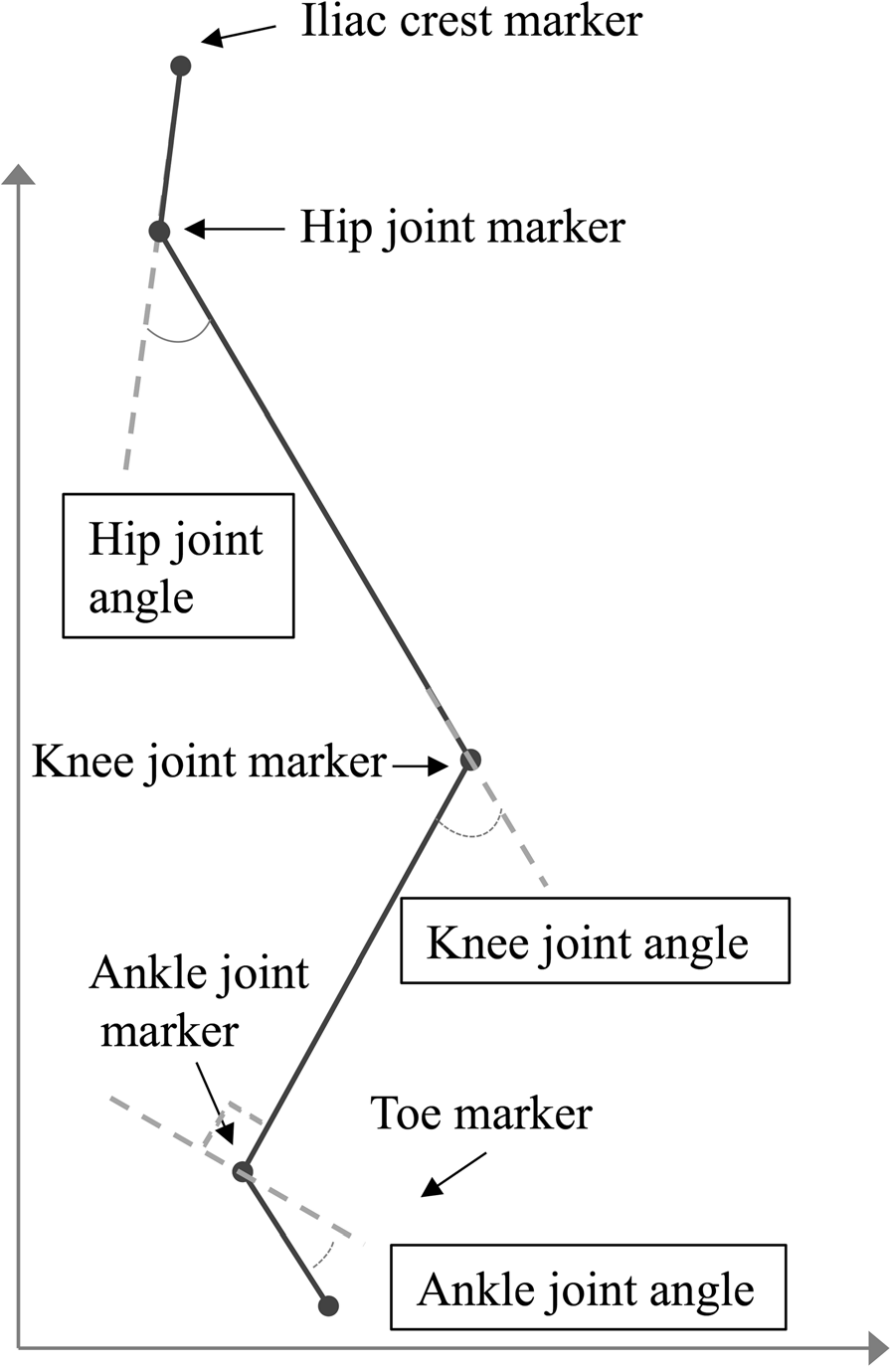
Simplified model for joint angle measurement. The joint angles were calculated as the angles defined by the joint markers on the sagittal plane

The joint angles of the hip, knee, and ankle were represented by the trunk-thigh angle, thigh-crus angle, and crus-foot angle, respectively, which were defined by the marker positions in the sagittal plane, as follows: the hip joint angles were defined as the angles between the iliac crest marker, hip marker, and knee marker; the knee joint angles were determined from the angles between the hip marker, knee marker, and ankle marker; and the ankle joint angles were calculated from the angles between the knee marker, ankle marker, and toe marker (Fig. 4).

**Fig. 5:**
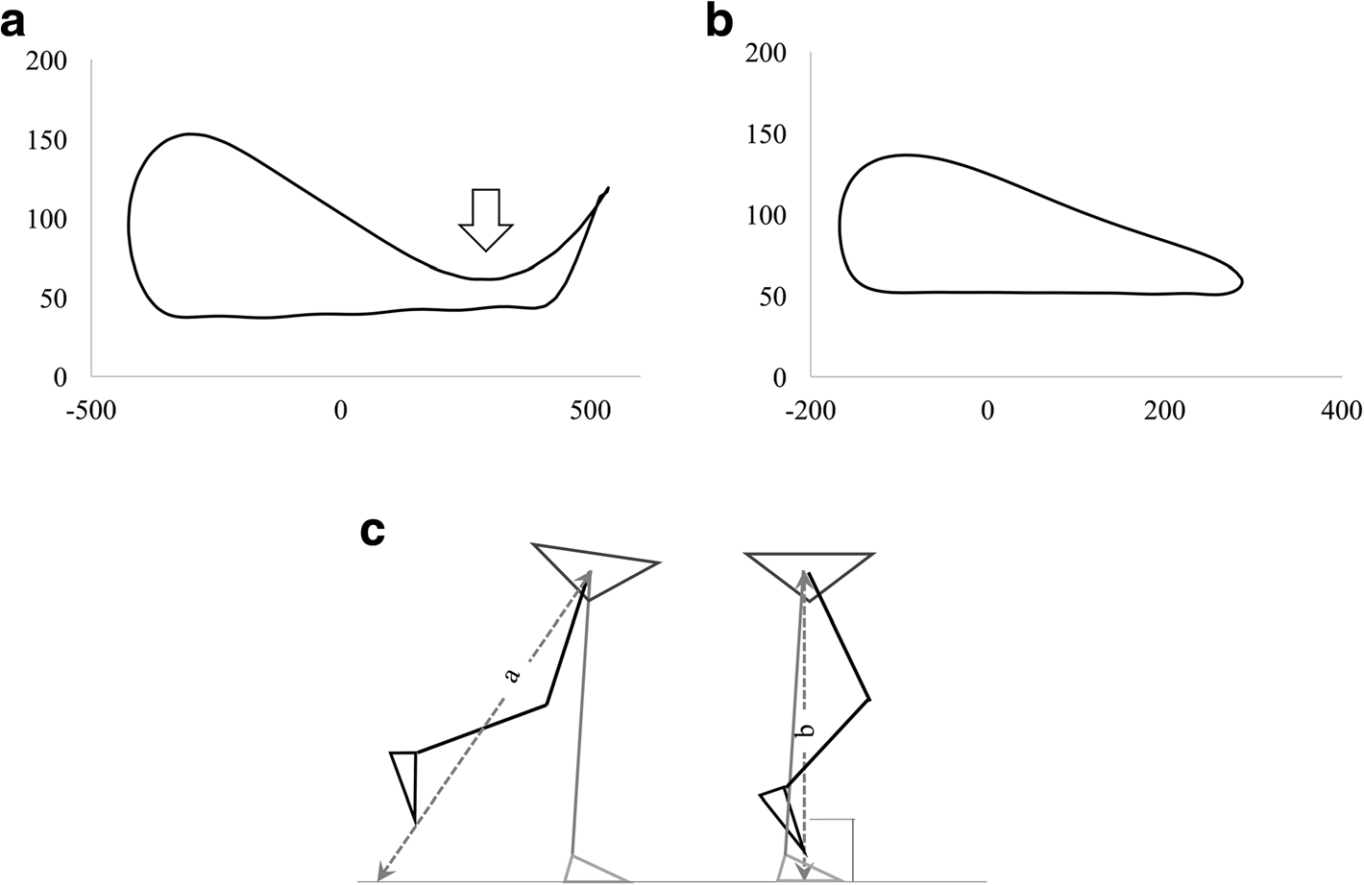
Minimum toe clearance in healthy subjects and hemiparetic patients. A,B: The trajectory of the toe on the treadmill. **a** representative toe trajectory of healthy subjects. The white arrow indicates the point of minimum toe clearance. **b** Toe trajectory of hemiparetic patients without a point of minimum toe clearance. **c** Schematic diagram showing the length of hip–toe–ground line during the swing phase. The hip–toe–ground distance would be shortest at the time when the toe crosses the vertical line through the hip (a > b)

Unlike prior studies that commonly used minimum toe clearance (MTC) as a representation of toe clearance ability during mid-swing [32, 33], the present study evaluated toe clearance at the time when the toe crosses the vertical line from the hip marker. The vertical trajectory of the toe, which follows a downward-pointing curve during mid-swing in healthy individuals, was absent in some of our post-stroke patients, and instead showed an upward-pointing curve during swing phase trajectory which precluded calculation of the MTC (Fig. 5a,b). For this reason, we identified toe clearance at the time when the toe passed directly underneath the hip marker, when the hip–floor distance through the toe (dotted line) would be the shortest without hip elevation (Fig. 5c). This timing is also ideal for calculating the impact of limb shortening and compensatory movement because the hip and the toe markers are both in the same frontal plane at this time. Therefore, the impact of the circumduction on toe movement in the vertical axis could be easily calculated independently from the impact of other toe clearance-related limb movements.

Subgroup analyses were performed to compare the effect of AFOs between subacute and chronic stroke patients, and between tAFOs and APS-AFOs.

Statistical analyses were performed using SPSS version 19.0 (SPSS, Chicago, IL) and JMP 12 (SAS Institute Inc. Cary, NC, USA). Descriptive statistics were used to describe patients’ demographic characteristics. To enable further analysis of parameters, the mean and standard deviation (SD) were presented. The changes in gait parameters were evaluated using the paired t-test. The Student’s t-test (unpaired t-test) was used to compare the effect of AFOs on the alterations of joint displacement between two subgroups: stroke patients in the subacute vs the chronic stage, and patients with a tAFO vs an APS-AFO. The goodness of fit was computed using the Shapiro-Wilk test, and showed normal distribution of data. Values of *P* < 0.05 were considered to indicate statistically significant differences.

## Results

Patient characteristics and comfortable self-selected speeds used at walking trial both with and without AFO in each participant are presented in Table 1.

**Table 1:** Patient characteristics and self-selected speed used at walking trial with and without AFO

ID	Age (years)	Gender	Diagnosis	Affected side	SIAS-LE	TAO (days)	Self-selected speed on treadmill (km/hr)	Type of AFO
					/15			
1	58	M	Hemorrhage	L	5	2313	1.0	APS-AFO
2	41	M	Hemorrhage	L	12	32	1.7	tAFO
3	58	M	Hemorrhage	R	10	57	1.7	tAFO
4	80	M	Hemorrhage	R	9	104	1.8	APS-AFO
5	60	M	Infarction	R	8	765	1.9	APS-AFO
6	21	F	Hemorrhage	R	8	2439	2.0	tAFO
7	43	F	Hemorrhage	L	8	38	2.2	APS-AFO
8	18	M	Hemorrhage	L	8	81	2.3	APS-AFO
9	15	F	Hemorrhage	R	11	1809	2.3	tAFO
10	58	M	Hemorrhage	R	9	413	2.4	tAFO
11	74	M	Hemorrhage	L	11	876	2.4	APS-AFO
12	52	F	Hemorrhage	R	11	543	2.5	tAFO
13	55	M	Hemorrhage	L	10	1579	2.5	tAFO
14	38	F	Hemorrhage	L	8	2260	2.5	tAFO
15	79	F	Infarction	L	10	84	2.6	tAFO
16	53	M	Infarction	L	9	322	2.6	tAFO
17	64	M	Hemorrhage	L	11	1768	2.6	APS-AFO
18	14	M	Infarction	L	10	233	2.7	APS-AFO
19	53	M	Hemorrhage	R	12	47	2.8	APS-AFO
20	47	M	Infarction	R	10	80	2.8	APS-AFO
21	47	M	Hemorrhage	L	8	341	2.8	tAFO
22	43	M	Infarction	R	12	73	3.3	tAFO
23	26	M	Hemorrhage	L	12	81	4.0	APS-AFO
24	33	M	Hemorrhage	R	10	200	4.2	APS-AFO

*AFO* Ankle–foot orthosis, *APS-AFO* Adjustable posterior strut AFO, *F* Female, *M* Male, *No* Number, *TAO* Time after onset, *tAFO* Thermoplastic AFO

**Fig. 6:**
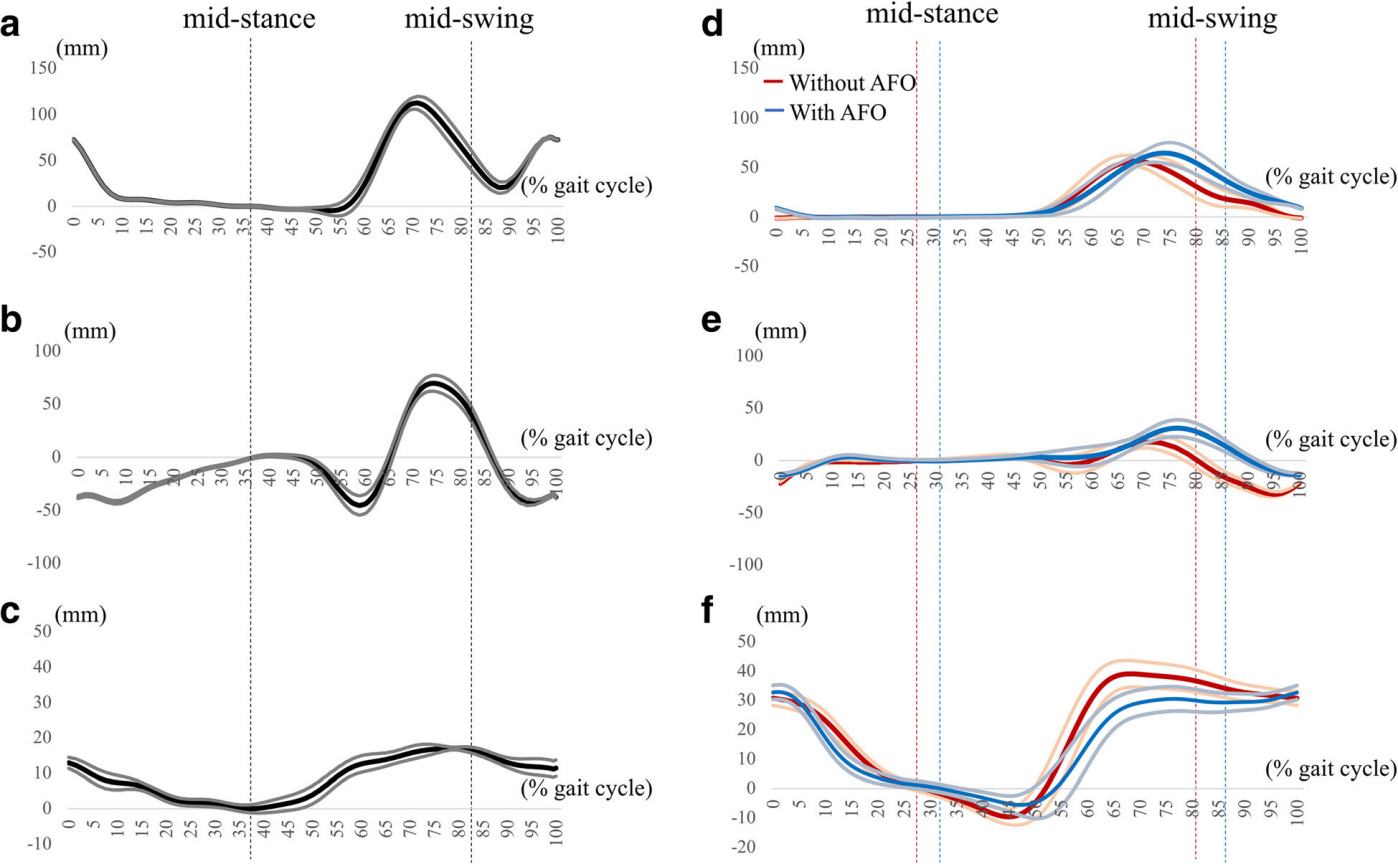
Representative trajectories of gait indices: The representative trajectories (mean ± SD) of 1) Toe clearance (**a**, **d**), 2) Shortening of hip-toe distance (**b**, **e**) and 3) Paretic hip elevation due to pelvic obliquity (**c**, **f**) in a healthy subject (**a**-**c**) and a hemiparetic patient with (Blue line) and without (Red line) AFO(**d**-**f**)

Representative trajectories of toe clearance, shortening of hip-toe distance, and paretic hip elevation due to pelvic obliquity are presented in Fig. 6.

**Fig. 7:**
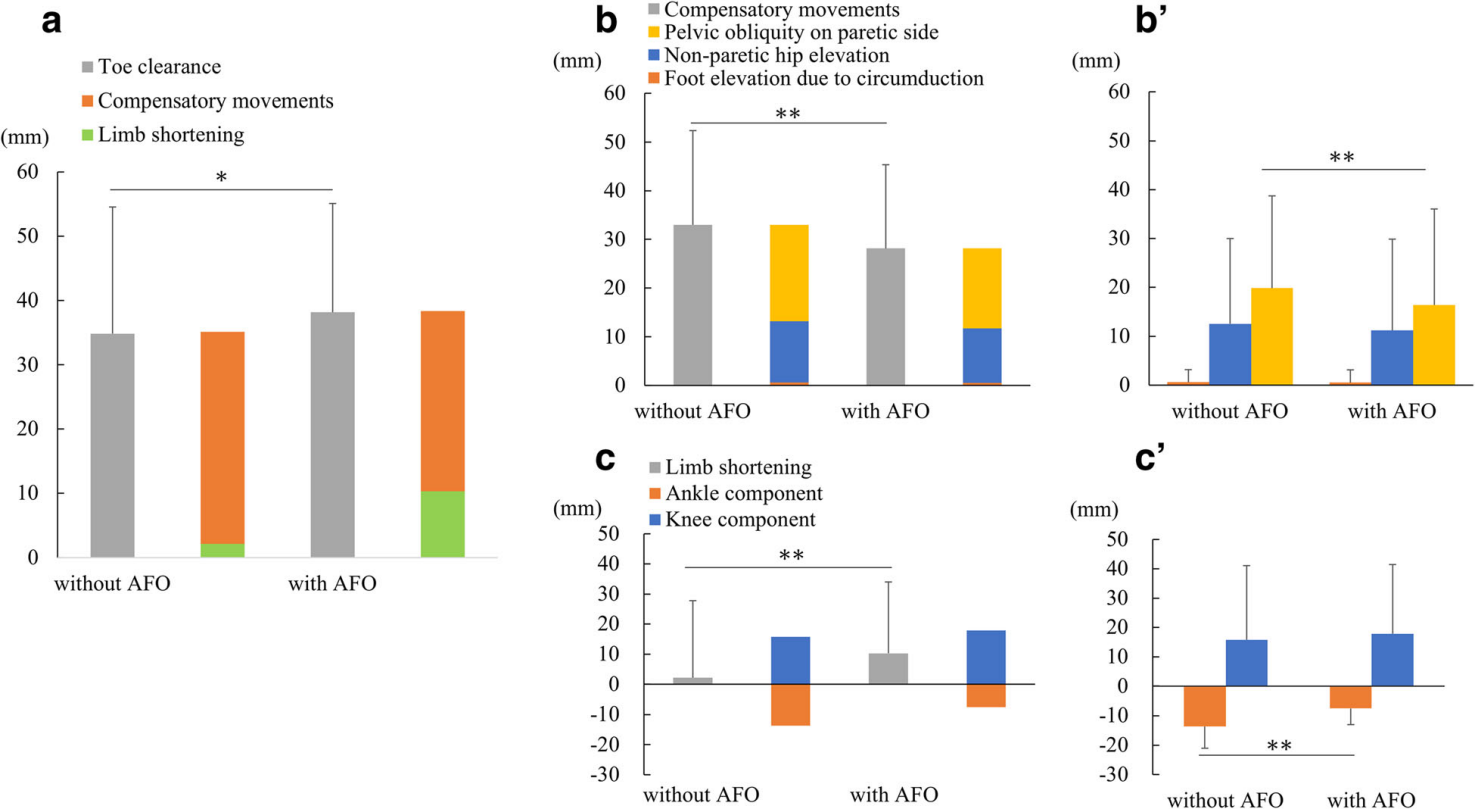
Toe clearance and the impact of gait-related movements. The impacts on toe clearance by each movement were calculated as the vertical displacement (mm) due to each movement. The toe clearance consists of components of limb shortening and compensatory movements (**a**). The effect of an ankle-foot orthosis (AFO) on compensatory movements (**b**, **b’**). The impacts of compensatory movements and its components of compensatory movements (pelvic obliquity on paretic side, non-paretic hip elevation, and foot elevation due to circumduction) were quantified (**b**, **b’**). The effect of limb shortening, comprising two components, was quantified. The ankle component and knee component were estimated separately to determine the effect of the AFO (**c**, **c’**)

The gait indices (mid-swing–mid-stance) are summarized in Fig. 7. The toe clearance was significantly increased by the use of an AFO (Fig. 7a: 33.9 ± 19.6 vs. 37.6 ± 16.9, *p* = 0.038). The toe clearance could be divided into two parts: the limb shortening and compensatory movements. The compensatory movements were significantly decreased by the use of the AFO (Fig. 7b; 33.0 ± 19.4 vs. 28.1 ± 17.2: *p* = 0.001), whereas the limb shortening was increased (Fig. 7c; 2.2 ± 25.6 vs. 10.3 ± 23.7: *p* < 0.001). Among the three components of compensatory movements (pelvic obliquity on paretic side, non-paretic hip elevation, and foot elevation due to circumduction), a significant difference was noted with and without the AFO for the pelvic obliquity on the paretic side (19.8 ± 18.9 vs. 16.4 ± 19.7, *p* = 0.003), and a weak tendency was noted for a reduction in the non-paretic hip elevation by the AFO (12.6 ± 17.4 vs. 11.2 ± 18.6, *p* = 0.234). No difference was observed in the foot elevation due to circumduction (Fig. 7b’). The limb lengthening by ankle movement was significantly decreased by the use of AFO (− 13.6 ± 7.4 vs. -7.6 ± 5.4, *p* < 0.0001), contributing to limb shortening. No significant change was observed in limb shortening due to knee movement (15.8 ± 25.3 vs. 17.9 ± 23.6, *p* = 0.183; Fig. 7c’).

**Fig. 8:**
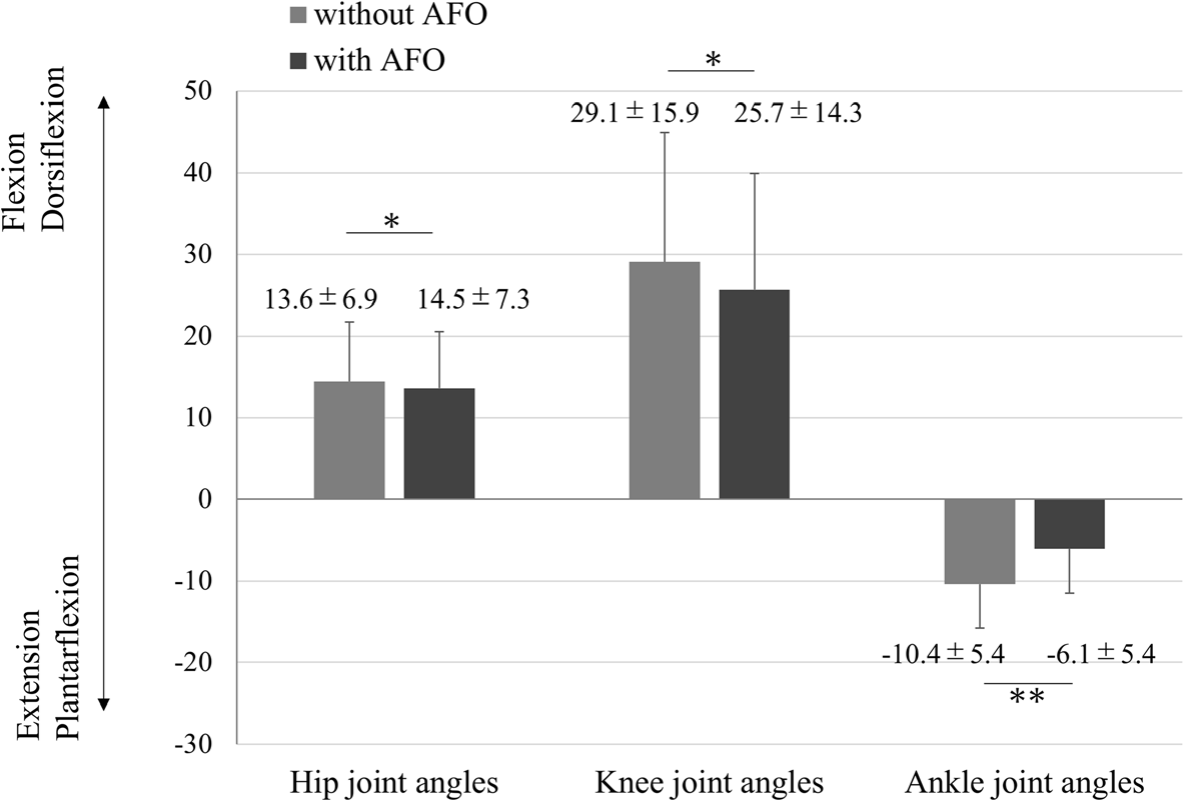
Joint angle changes on the paretic limb in walking trials performed with and without ankle-foot orthoses (AFOs). The joint angle changes from mid-stance to mid-swing on a paretic limb. Error bar: SD *:< 0.05, **:< 0.01. *P* values were obtained by a paired t-test

The joint angle changes on the paretic limb in walking trials performed with and without AFOs was shown in Fig. 8. There were significant quantitative differences between trials performed with AFOs and those performed without AFOs in the decreases in hip flexion (13.6 ± 6.9 (with) vs 14.5 ± 7.3 (without), *p* = 0.019), knee flexion (25.7 ± 14.3 (with) vs 29.1 ± 15.9 (without), *p* = 0.001), and ankle plantar flexion (6.1 ± 5.4 (with) vs 10.4 ± 5.4 (without),*p* = 0.007) during the swing phase.

The subgroup analyses regarding the effect of AFOs on kinematic components revealed no significant differences in any of the measured indices in subacute vs chronic stroke patients, or in tAFOs vs APS-AFOs.

## Discussion

The present study confirmed the positive influence of AFOs on functional limb shortening, with resulting improvements in toe clearance and compensatory movement. The average toe elevation in hemiparetic gait was enhanced by the use of AFOs, irrespective of the stage of hemiparesis and/or the type of AFO used. Using an AFO resulted in an increase in ankle dorsiflexion during the swing phase and a subsequent decrease in compensatory movements, which was consistent with previous studies [17, 18, 34]. Furthermore, the present findings manifested the simple relationship between the changes in limb shortening and compensatory movements to achieve toe clearance, by breaking down each movement into the actual impact on toe clearance. As shown in this study, the toe clearance was increased by the use of AFOs (33.9 vs. 37.6, *P* = 0.038), due to the increase in vertical gain by limb shortening (2.2 vs. 10.3, *p* < 0.001). The compensatory movements account for the rest. This type of visualization could be useful for monitoring the beneficial effects of AFOs.

Improved joint motions and decreased compensatory movement when using AFOs could potentially contribute to efficient gait and promote walking activity in hemiparetic patients.

**Fig. 9:**
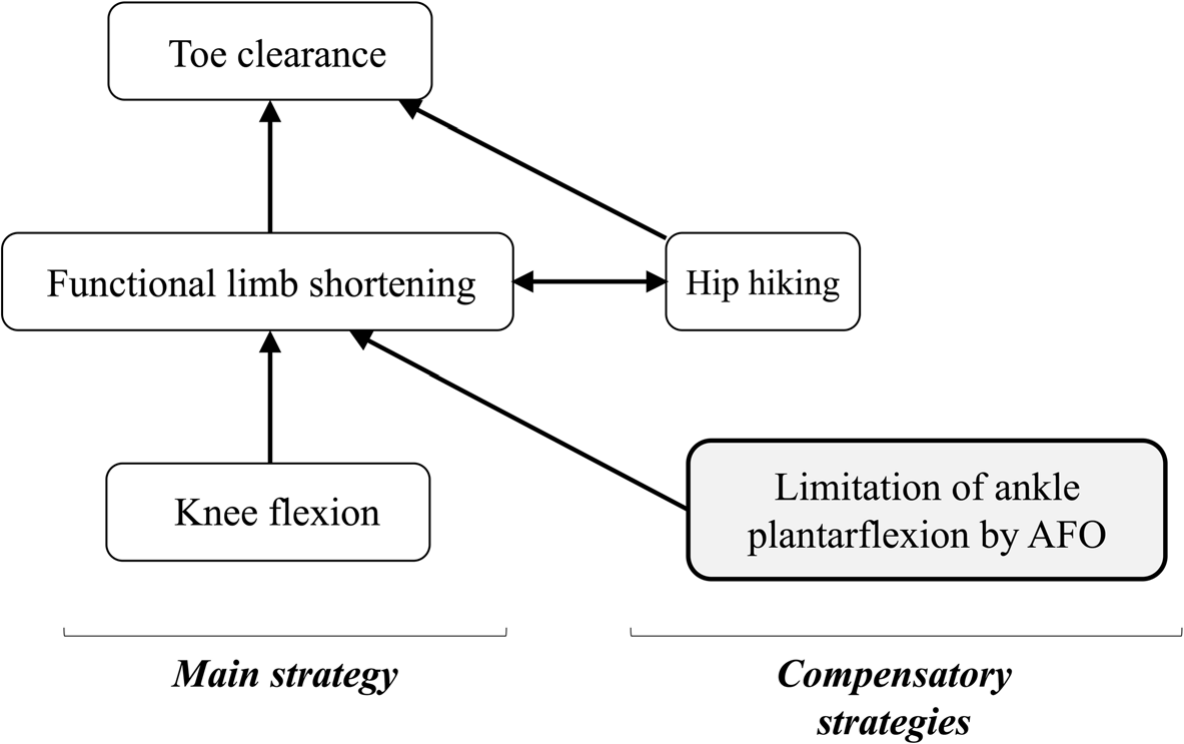
The possible discrepancy between the knee flexion angle and its impact on limb shortening. The positional relationships between the hip, knee, ankle and toe markers at the mid-swing(**a**,**b**). Reduced knee flexion angle (θ1 > θ2) could lead to increased limb shortening (L1 > L2), in cases where the ankle angles are fixed

The degree of limb shortening is mainly determined by the degree of knee flexion, especially in the healthy subjects or patients with mild paresis [10]. Conversely, AFOs achieve limb shortening due to the mechanical property that limits ankle plantarflexion, which could be referred to as another compensatory approach to improve limb shortening. The present and previous findings indicate that toe clearance in hemiparetic patients with the use of AFOs could be achieved by the following mechanism. First, decreased knee flexion in hemiparetic gait may be compensated for by the AFO’s effect on decreasing ankle plantar flexion and subsequently maintaining the optimal hip-toe distance. Second, hip hiking (as a compensatory gait pattern) is consequently minimized because of acquired optimal limb shortening, as shown in the present study. The interrelationship of relevant gait indices are hypothesized as shown in Fig. 9.

**Fig. 10:**
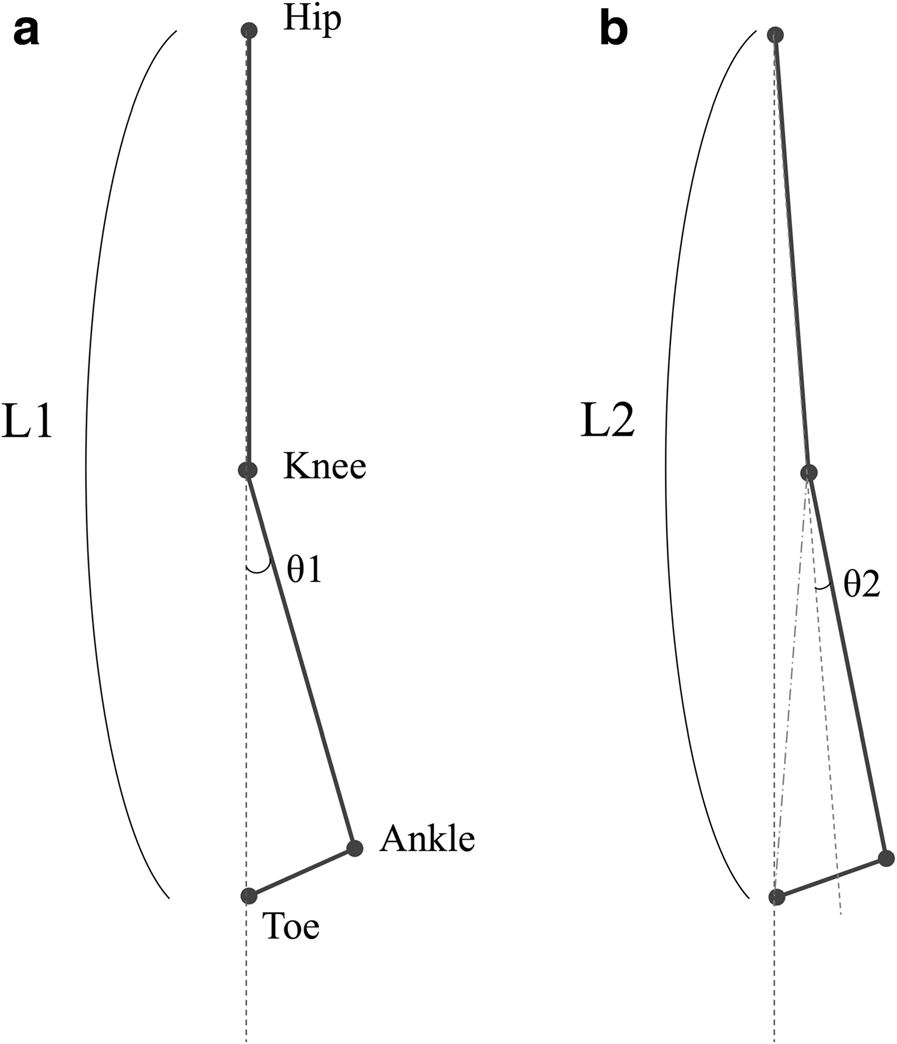
Diagram illustrating the relationships between toe clearance, functional limb shortening, and compensatory movements

Decreased knee flexion may be considered a negative effect of AFOs on the hemiparetic gait. The possible explanations for this include the following: First, ankle plantar flexion that has been decreased by AFO decreases limb lengthening, so the patient might not need to flex the knee as much to shorten the limb, because the same toe clearance can be achieved with less effort. In fact, in the present study, the use of AFO decreased limb lengthening due to ankle plantar flexion (Fig. 7c). In addition, the weight of the orthosis might influence knee flexion by making it more difficult for the paretic limb to flex the knee against gravity. Another possibility is the discrepancy between the knee-flexion angle and the effect of the knee flexion, which is seen when the knee-flexion angle is very small. In this case, slight knee flexion does not merit the toe clearance. For example, if the hip-knee-toe markers were aligned on the vertical line (Fig. 10a) and the ankle angle was fixed, the knee flexion could lengthen the hip-toe distance (Fig. 10b). In this situation, a discrepancy would arise between the knee-flexion angle and the limb shortening; a small knee flexion may not have a beneficial effect on limb shortening. The present study identified a conflict between the effect of AFO on reducing knee flexion and the relative increase in limb shortening due to knee movement (Figs. 7c and 8), which might have been influenced by this paradoxical relationship between joint angle and limb shortening.

In addition, the effect of AFO use on knee angles itself could have influenced by the different characteristics of the participant groups. A previous study showed an increase in knee flexion by AFO use in hemiplegic patients; Gatti et al. reported a positive effect of AFO use on knee flexion angle (26.316 without AFO vs. 30.785 with AFO) in the patient group with paresis scores of 4–5 on the Scandinavian Stroke Scale, which is milder paresis than in the present study [35].The effect of AFO use on knee movements should be further investigated, including whether the reduction in knee flexion has a negative impact on the recovery of knee movement.

The use of an AFO had a weak tendency to reduce the elevation of the non-paretic limb. Non-paretic hip elevation may occur in the following situations. First, it can occur if there is unusual elevation of the pelvis at the mid-swing of the paretic limb. This can be achieved either by ankle plantar flexion or by over-extension of the knee at the mid-stance of the non-paretic limb. However, the data did not evidence either of these causes.

Second, this can occur when the vertical position of the pelvis is lowered at the mid-stance of the paretic limb and elevated during the swing phase of the paretic limb. The lowered vertical position of the pelvis can occur due to the knee flexion of the paretic limb or the drop of the non-paretic side of the pelvis (Trendelenburg’s sign). In this case, the non-paretic hip drop showed significant correlation with the extent of the non-paretic hip elevation, while paretic knee flexion did not, indicating that the lowered pelvic position caused by the drop of the non-paretic side of the pelvis during the stance phase of the paretic limb was the main reason for the non-paretic hip elevation during the swing phase of the paretic limb. There was a weak, non-significant tendency of this drop of the pelvis to be decreased by the use of AFO (Additional file 4: Figure S3). The AFO effect on this non-paretic limb elevation must be confirmed in studies that have larger samples.

The present study clarified the real clinical picture of AFO-affected gait patterns, contributing a deepened understanding of the systematically balanced relationship between the enhancement of paretic limb clearance and the ability to reduce compensatory movement during the swing phase. Recognition and quantification of the interrelationships among the changes in joint kinematics, joint displacement, functional limb shortening, and the actual impacts on toe clearance would contribute to the comprehensive understanding of the effects of intervention and help clinicians to develop strategies to guide patients toward improving the advancement of the paretic limb during the swing phase. For example, when toe clearance is insufficient due to paresis, clinicians could consider facilitating improvements in the ability to effect limb shortening; otherwise, they could prepare AFOs for that purpose. If the effects are insufficient, compensatory movements could be encouraged. Conversely, once the clinician can confirm that the patient has gained sufficient toe clearance, the focus of the intervention could be redirected to the reduction of compensatory movements. With the use of gait analysis systems, we can quantify the actual impact and monitor the effect of each intervention and set goals for the patient. This will require further quantitative investigations in future studies for development, but this structured quantification may enable tailor-made rehabilitation training for individual patients. In addition to facilitating toe clearance in the swing phase, AFOs assist in other aspects of gait such as gait stability during the stance phase. Further analysis of the effect of AFOs should be encouraged to further the comprehensive understanding of the holistic effect of AFO use.

This study had a number of limitations. First, all included patients were able to independently ambulate on the treadmill without gait aids or orthoses. The findings may not be representative of those with severe hemiparesis. Further analysis of patients with more impaired gait abilty would facilitate further understanding of the effect of AFOs. Second, there was a relatively small sample population with heterogeneity. However, subgroup analyses found that the type of AFO and stage of hemiparesis did not significantly affect the kinematic parameters. Third, there was a small number of markers compared with commonly-used marker sets such as plug-in gait and Helen Hayes [36, 37], and the joint angles were indirectly estimated from the positions of the joint markers in the sagittal plane. We consider that this would not be critical enough to negate the present results, as the validity of measuring abnormal gait patterns, including the degree of joint angle movements, using this simple marker set has been shown previously [29, 38–40]. However, the limitations of this simplified method should be further confirmed in future studies.

## Conclusion

The present study quantified the impact of AFOs on toe clearance strategy in hemiparetic patients, and investigated the interrelationships between movement parameters. The results offer an insight into the effects of AFOs on the overall biomechanism of hemiparetic gait during the swing phase. Furthermore, these attempts to quantify and visualize the gait strategy could contribute to the clinical judgment and decision-making regarding rehabilitation strategy, which will help achieve better outcomes in rehabilitation practice.

## Additional files

Additional file 1: **Figure S1.** Adjustable posterior strut ankle–foot orthosis (APS-AFO). (TIFF 22671 kb)

Additional file 2: Supplemental methods and results. The details of additional experiment for clarifying measurement error. (DOCX 16 kb)

Additional file 3: **Figure S2.** Experimental setting for clarifying measurement error. A: A 1-m-long aluminum bar with four markers on it. B-D: The participant held the aluminium bar in three ways while walking; parallel to his torso, parallel to the sagittal and horizontal planes, or parallel to the coronal and horizontal planes. (PNG 1540 kb)

Additional file 4: **Figure S3.** Correlation among non-paretic hip elevation (at mid-swing), paretic knee flexion, and non-paretic hip drop at paretic mid-stance. A. Correlation between the non-paretic hip elevation at the paretic mid-swing and the paretic knee flexion at the paretic mid-stance. The correlation coefficient was 0.10 (*p* = 0.80). B. Correlation between the non-paretic hip elevation at the paretic mid-swing and the non-paretic hip drop at the paretic mid-stance. The correlation coefficient was − 0.60 (*p* < 0.01). C. Comparison of the non-paretic hip drop at the paretic mid-stance with and without AFO (*p* = 0.23). (PNG 1140 kb)

## Abbreviations

AFOs: Ankle–foot orthoses
APS-AFO: Adjustable posterior strut ankle–foot orthosis
tAFO: Thermoplastic ankle–foot orthosis
TAO: Time after onset
