# Supplementary figures and images for: The impact of ankle–foot orthoses on toe clearance strategy in hemiparetic gait: a cross-sectional study
Source: J Neuroeng Rehabil. 2018 May 23;15:41. doi: 10.1186/s12984-018-0382-y (PMC5966858; doi:10.1186/s12984-018-0382-y)

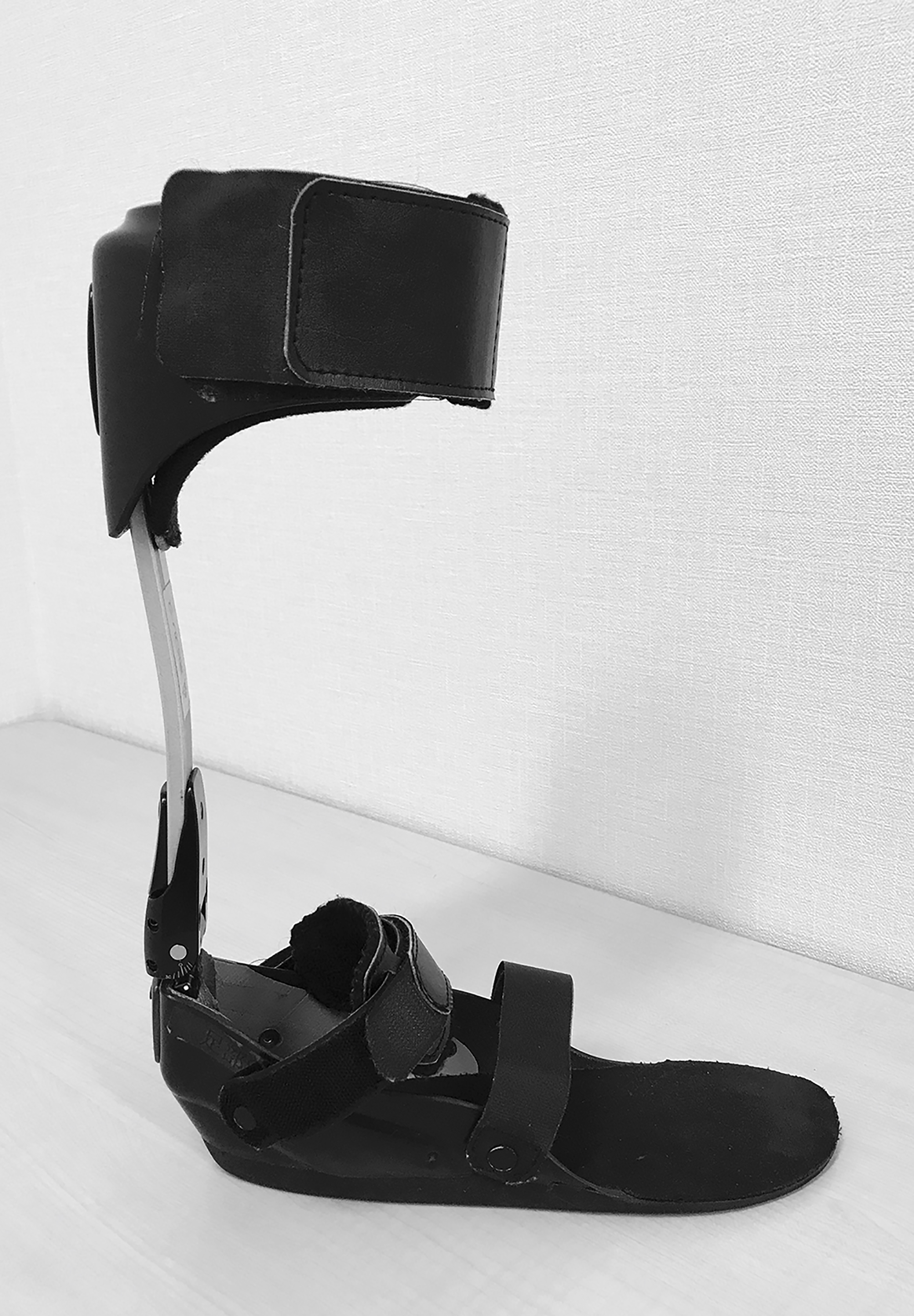

Supplement: Supplementary file 1 — Figure S1. Adjustable posterior strut ankle–foot orthosis (APS-AFO). (TIFF 22671 kb) [file 12984_2018_382_MOESM1_ESM.tiff]

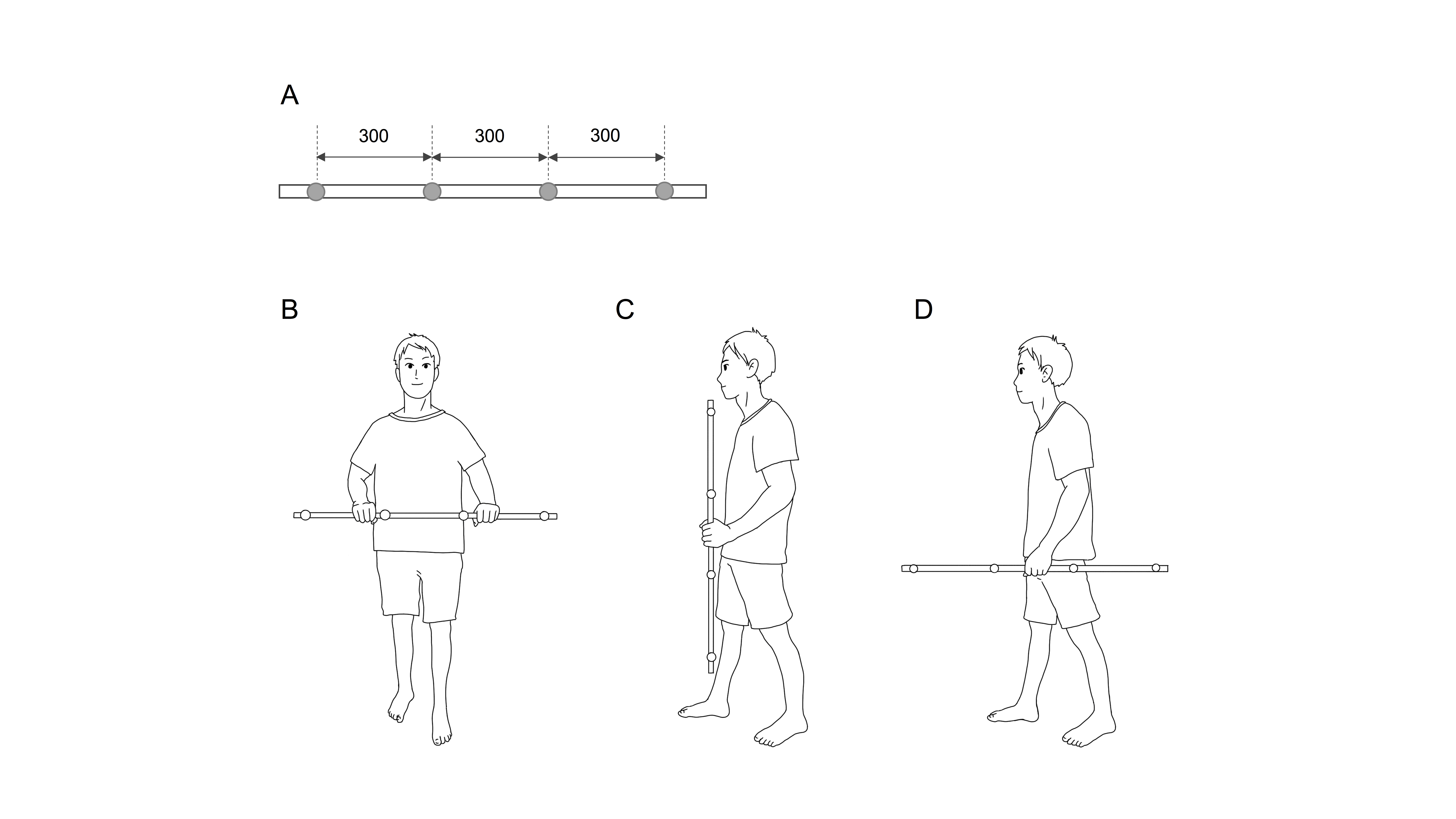

Supplement: Supplementary file 3 — Figure S2. Experimental setting for clarifying measurement error. A: A 1-m-long aluminum bar with four markers on it. B-D: The participant held the aluminium bar in three ways while walking; parallel to his torso, parallel to the sagittal and horizontal planes, or parallel to the coronal and horizontal planes. (PNG 1540 kb) [file 12984_2018_382_MOESM3_ESM.png]

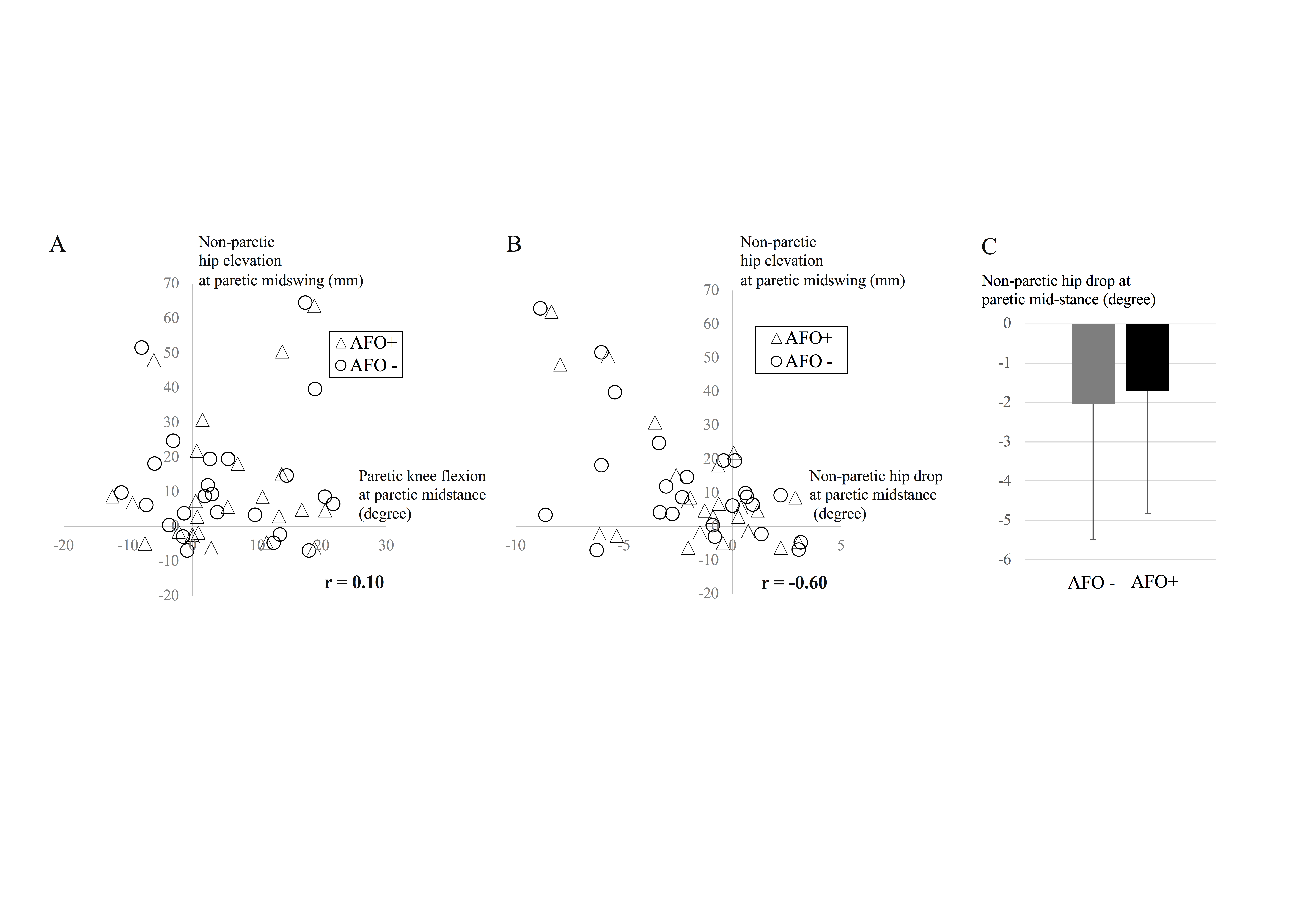

Supplement: Supplementary file 4 — Figure S3. Correlation among non-paretic hip elevation (at mid-swing), paretic knee flexion, and non-paretic hip drop at paretic mid-stance. A. Correlation between the non-paretic hip elevation at the paretic mid-swing and the paretic knee flexion at the paretic mid-stance. The correlation coefficient was 0.10 (p = 0.80). B. Correlation between the non-paretic hip elevation at the paretic mid-swing and the non-paretic hip drop at the paretic mid-stance. The correlation coefficient was − 0.60 (p < 0.01). C. Comparison of the non-paretic hip drop at the paretic mid-stance with and without AFO (p = 0.23). (PNG 1140 kb) [file 12984_2018_382_MOESM4_ESM.png]
