# Supplementary material for: The impact of ankle–foot orthoses on toe clearance strategy in hemiparetic gait: a cross-sectional study
Source: J Neuroeng Rehabil. 2018 May 23;15:41. doi: 10.1186/s12984-018-0382-y (PMC5966858; doi:10.1186/s12984-018-0382-y)
Supplement: Supplementary file 2 — Supplemental methods and results. The details of additional experiment for clarifying measurement error. (DOCX 16 kb) [file 12984_2018_382_MOESM2_ESM.docx]

Supplemental methods

We performed experiment with a modified protocol based on the evaluation protocol of measurement error of three-dimensional motion analysis systems developed by The Clinical Gait Analysis Forum of Japan [1].

A 29 year-old male with no movement disorders participated in this experiment. For the measurement, we used a 1 m long aluminum bar, on which four markers were attached 300 mm apart (Supplemental figure 2A). The exact distance between markers was measured with a slide caliper. The subject walked on the treadmill at a speed of 3.6 km/h and a cadence of 100 steps/min, while holding the aluminum bar in three positions: parallel to his torso, parallel to the sagittal and horizontal planes, and parallel to the coronal and horizontal planes (Supplemental figure 2B-D). Each measurement lasted for 20 seconds and the sampling frequency was 60Hz. After the measurements, the mean value and standard deviation of the distance between the adjacent markers and the mean value of the absolute error were calculated for each task.

1. Ehara, Y., Fujimoto, H., Miyazaki, S., Mochimaru, M., Tanaka, S., & Yamamoto, S. (1997). Comparison of the performance of 3D camera systems II. *Gait & Posture*, *5*(3), 251-255.
2. Carse, B., Meadows, B., Bowers, R., & Rowe, P. (2013). Affordable clinical gait analysis: An assessment of the marker tracking accuracy of a new low-cost optical 3D motion analysis system. *Physiotherapy*, *99*(4), 347-351.

Supplemental results:

The results are shown in supplemental table1. The averaged absolute error ranged from 0.5–1.7mm in the antero-posterior axis, 1.6–1.7mm in the vertical axis, and 1.5–2.4mm in the lateral axis (Supplemental table 1).

Supplemental table 1: Measurement error for each axis

|  | Average ±SD (mm) |  | Averaged absolute error (mm) |
| --- | --- | --- | --- |
| Antero-posterior axis |  |  |  |
| Anterior (a) | 299.8±0.9 |  | 0.8±0.5 |
| Middle (b) | 299.8±0.6 |  | 0.5±0.4 |
| Posterior (c) | 301.6±0.8 |  | 1.7±0.7 |
|  |  |  |  |
| Vertical axis |  |  |  |
| Upper (g) | 301.6±0.8 |  | 1.7±0.7 |
| Middle (h) | 298.4±0.7 |  | 1.6±0.7 |
| Lower (i) | 298.4±0.8 |  | 1.6±0.7 |
|  |  |  |  |
| Lateral axis |  |  |  |
| Right (d) | 297.6±0.6 |  | 2.4±0.6 |
| Middle (e) | 297.6±0.7 |  | 2.4±0.6 |
| Left (f) | 298.5±0.6 |  | 1.5±0.6 |
|  |  |  |  |
|  | (True value: 300) |  |  |
